# Identification of CEACAM5 as a stemness-related inhibitory immune checkpoint in pancreatic cancer

**DOI:** 10.1186/s12885-022-10397-7

**Published:** 2022-12-09

**Authors:** Haojun Shi, Yiusing Tsang, Yisi Yang

**Affiliations:** 1 Department of General Surgery, Ruijin Hospital, Shanghai Jiao Tong University School of Medicine, Shanghai, China; 2 Shanghai Institute for Endocrine and Metabolic Diseases, Ruijin Hospital, Shanghai Jiao Tong University School of Medicine, Shanghai, China; 3 Graduate School of Asia-Pacific Studies, Waseda University, Tokyo, Japan

**Keywords:** Pancreatic cancer, Cancer stemness, CEACAM5, Immune checkpoint, Immunotherapy

## Abstract

**Background:**

Immunotherapy has emerged as a new cancer treatment modality. However, tumour heterogeneity can diminish checkpoint blockade response and shorten patient survival. As a source of tumour heterogeneity, cancer stem cells act as an indispensable reservoir for local recurrence and distant metastasis. Thus, precision immunotherapy targeting tumour heterogeneity requires a comprehensive understanding of cancer stem cell immunology. Our study aimed to identify stemness-related inhibitory immune checkpoints and relevant regulatory pathways in pancreatic cancer.

**Methods:**

Pancreatic cancer-specific datasets in The Cancer Genome Atlas and the Cancer Therapeutics Response Portal were collected for in-depth bioinformatic analysis. Differentially expressed genes between pancreatic cancers with high and low stemness index (mRNAsi) scores were compared to screen out inhibitory immune checkpoints. Survival analysis was used to predict the prognostic value of immune checkpoint plus immune infiltrate in patients with pancreatic cancer. The expression of stemness-related immune checkpoint across immune subtypes of pancreatic cancer was detected and gene set enrichment analysis was performed to figure out the relevant regulatory signallings.

**Results:**

The abundance of cancer stemness predicted a low immunotherapy response to pancreatic cancer. The inhibitory immune checkpoint CEACAM5 that was enriched in pancreatic cancers with high mRNAsi scores also exhibited a strong correlation with invasive cell-enriched signature and Msi^+^ tumour-initiating cell-enriched signature. Levels of CEACAM5 expression were higher in the interferon-γ dominant immune subtype of pancreatic cancers that are characterized by high M1 macrophage infiltration. The patient group with high levels of CEACAM5 expression had a high risk of poor overall survival, even if accompanied by high infiltration of M1 macrophages. Furthermore, prostanoid and long-chain unsaturated fatty acid metabolic processes showed a significant association with cancer stemness and CEACAM5 expression.

**Conclusions:**

Our findings suggest that CEACAM5 is a candidate stemness-related innate immune checkpoint in pancreatic cancer, and is potentially regulated by prostanoid and long-chain unsaturated fatty acid metabolic processes. Immune checkpoint blockade of CEACAM5, which synergizes with inhibition of those regulatory pathways, may improve the efficacy of precision immunotherapy targeting tumour heterogeneity caused by cancer stem cells.

**Supplementary Information:**

The online version contains supplementary material available at 10.1186/s12885-022-10397-7.

## Introduction

Pancreatic cancer continues to be the seventh leading cause of cancer death worldwide [1]. The 5-year survival rate for all stages combined is only 10%. Approximately 80–85% of pancreatic cancer are determined as unresectable at the time of diagnosis [2]. Those patients with either locally advanced or metastatic disease have to receive chemotherapy combinations, including FOLFIRINOX (5-fluorouracil, leucovorin, irinotecan, and oxaliplatin) and gemcitabine plus nab-paclitaxel, which merely prolong the survival by 6–12 months [3]. Although immunotherapy has emerged as a new cancer treatment modality, advances in pancreatic cancer have lagged far behind compared with melanoma [4] and non-small-cell lung carcinoma [5]. Early clinical trials on the efficacy of anti-CTLA-4 and anti-PD-L1 antibody in patients with advanced pancreatic cancer had disappointing results [6, 7]. Despite encouraging response rates of immune checkpoint agonist/antagonist-based regimens recently observed in patients with metastatic pancreatic cancer [8, 9], the limitations of current immunotherapy for pancreatic cancer are inevitably highlighted.

The resistance of pancreatic cancer to immunotherapy has been attributed to a low tumour mutational burden owing to a low incidence of mismatch repair deficiencies [10], a dense desmoplastic stroma caused by cancer-associated fibroblasts, and an immunosuppressive tumour microenvironment comprised of myeloid-derived suppressor cells, M2-like macrophages, and regulatory T cells [11]. However, a recent study demonstrates that intra-tumour heterogeneity characterized by clone numbers and their genetic diversity diminishes immune response and shortens patient survival [12]. As a source of intra-tumour heterogeneity, cancer stem cells residing at the top of the cancer hierarchy can self-renew and differentiate into diverse cell lineages found in malignant lesions [13]. More importantly, they may act as an indispensable reservoir for local recurrence and distant metastasis following radical surgery and systematic therapy [14]. For example, a subpopulation of CD133^+^CXCR4^+^ cancer stem cells was identified in the invasive front of pancreatic cancers. Eradication of this cancer stem cell pool abrogated the metastatic phenotype of pancreatic cancers [15]. α_v_β_3_-expressing cancer stem cells acquired high resistance to receptor tyrosine kinase inhibitors such as erlotinib by recruiting KRAS and RaIB and activating TBK1 and NF-κB [16]. To fuel tumour growth, CD9^+^ pancreatic cancer stem cells reallocated the glutamine transporter ASCT2 to the plasma membrane for enhancing glutamine uptake [17]. It is worth noting that the inconsistency between the tumour-initiating capacity of cancer stem cells in models with distinct immune responses reveals the immune privilege of cancer stem cells [18]. A subset of squamous cell carcinoma stem cells was found refractory to adoptive T cell transfer-based immunotherapy through acquiring CD80 to dampen cytotoxic T cell attack [19]. Cancer stem cells of head and neck squamous cell carcinoma upregulated CD276 to evade host immune responses of CD8^+^ T cells. CD276 blockade remodels tumor heterogeneity, reduces epithelial-mesenchymal transition, inhibits tumor growth and lymph node metastases [20]. Thus, it is also possible that pancreatic cancer stemness may be competent to drive tumour relapse due to immune checkpoint-mediated immune evasion. However, little is known about the immunological property of stemness-high pancreatic cancers.

Here, we used the datasets of pancreatic cancer from The Cancer Genome Atlas (TCGA), the Cancer Therapeutics Response Portal (CTRP) and the GEO dataset to identify stemness-related inhibitory immune checkpoints in pancreatic cancer. The interaction of pancreatic cancer stemness with the immune microenvironment and the consequent impact on patient prognosis were also evaluated to highlight the immune privilege of cancer stem cells within stemness-high pancreatic cancers. Given that downregulation of immune checkpoint expression and attenuation of cancer stemness can synergize with immune checkpoint blockade (ICB) in stemness-high pancreatic cancers, we also determined the regulatory pathways of cancer stemness and immune checkpoint and screened the drugs potentially sensitive to stemness-high pancreatic cancers with high CEACAM expression. In-depth knowledge of immunology in stemness-high pancreatic cancers will facilitate the development of efficient immunotherapies targeting pancreatic cancer stem cells to prevent and treat cancer recurrence and metastasis.

## Methods

### Sample collection and data processing

Gene expression data and corresponding clinical phenotypes of tumour samples were obtained from TCGA. RNA-seq data and clinical phenotypes for the TCGA cohorts were downloaded from the UCSC Xena project, including cholangiocarcinoma (CHOL, *n* = 36), colorectal adenocarcinoma (CRC, *n* = 620), oesophagal carcinoma (ESCA, *n* = 163), liver hepatocellular carcinoma (LIHC, *n* = 371), pancreatic adenocarcinoma (PAAD, *n* = 179), stomach adenocarcinoma (STAD, *n* = 375). RNA-seq data and drug activity for 22 pancreatic cancer cell lines were obtained from CTRP. The GEO dataset with accession number GSE21501 [21], which contains microarray and clinical data of 101 pancreatic cancer patients, was downloaded for validation of TCGA results. Because all data that our study used were from publicly available datasets TCGA, CTRP and GSE21501, no ethical approval was required to seek.

### Cancer stemness and immune landscape analysis

The stemness index (mRNAsi) was based on a one-class logistic regression (OCLR) machine learning algorithm and reflected transcriptomic stemness features of tumour cells [22]. The mRNAsi ranged from 0 to 1, and the closer mRNAsi was to 1, the more stem-like tumour cells. Tumour Immune Dysfunction and Exclusion (TIDE) algorithm is a computational method to predict cancer immunotherapy response [23]. Two distinct mechanisms were modelled to estimate tumour immune evasion, including dysfunction of infiltrating cytotoxic T lymphocytes and exclusion of cytotoxic T lymphocytes by immunosuppressive factors. A lower TIDE score indicates a slight chance of a satisfactory response to immunotherapy.

Deconvolution algorithms were used to quantify the cell composition of infiltrating immunocytes in tumour samples. MCP-counter deduced a global picture of stromal cells including T cells, CD8^+^ cells, cytotoxic lymphocytes, NK cells, B lineage, monocytic lineage, myeloid dendritic cells, neutrophils, endothelial cells and fibroblasts [24], while CIBERSORT estimated the abundance of 22 tumour-infiltrating lymphocytes in the tumour microenvironment more comprehensively [25]. Additionally, pancreatic cancers were divided into five immune subtypes with distinct therapeutic and prognostic implications for cancer management, including wounding healing, interferon-γ (IFN-γ), inflammatory, lymphocyte depleted, transforming growth factor-β (TGF-β) [26].

### Identification of differentially expressed genes and gene set enrichment analysis

Differentially expressed genes between low and high gene expression groups were screened out by the DESeq2 package. An adjusted *p*-value < 0.05 and |log2 (Fold Change)|> 1 was considered significant. Gene set enrichment analysis (GSEA) was conducted to analyse the pathways enriched in the high gene expression group to explore the underlying mechanisms. The screening conditions were |normalized enrichment score (NES)|> 1, nominal (NOM) *p*-value < 0.05 and FDR < 0.25.

### Correlation, mutually exclusivity and survival analysis

When performing comparison or correlation analysis in our study, we used log2(TPM + 1)-transformed data to normalize gene expression. Tumour purity adjustment was conducted to reduce the bias caused by the heterogeneous microenvironment. Mutations and CNAs of pancreatic cancer were used to identify mutually exclusive or co-occurrent events in the 37 selected immune checkpoint genes. An odds ratio indicated the likelihood, and a value over 2 meant a tendency toward co-occurrence. The results with a q-value < 0.05 were considered statistically significant.

## Results

### Association of cancer stemness with immune infiltrates across gastrointestinal cancer types

**Fig. 1:**
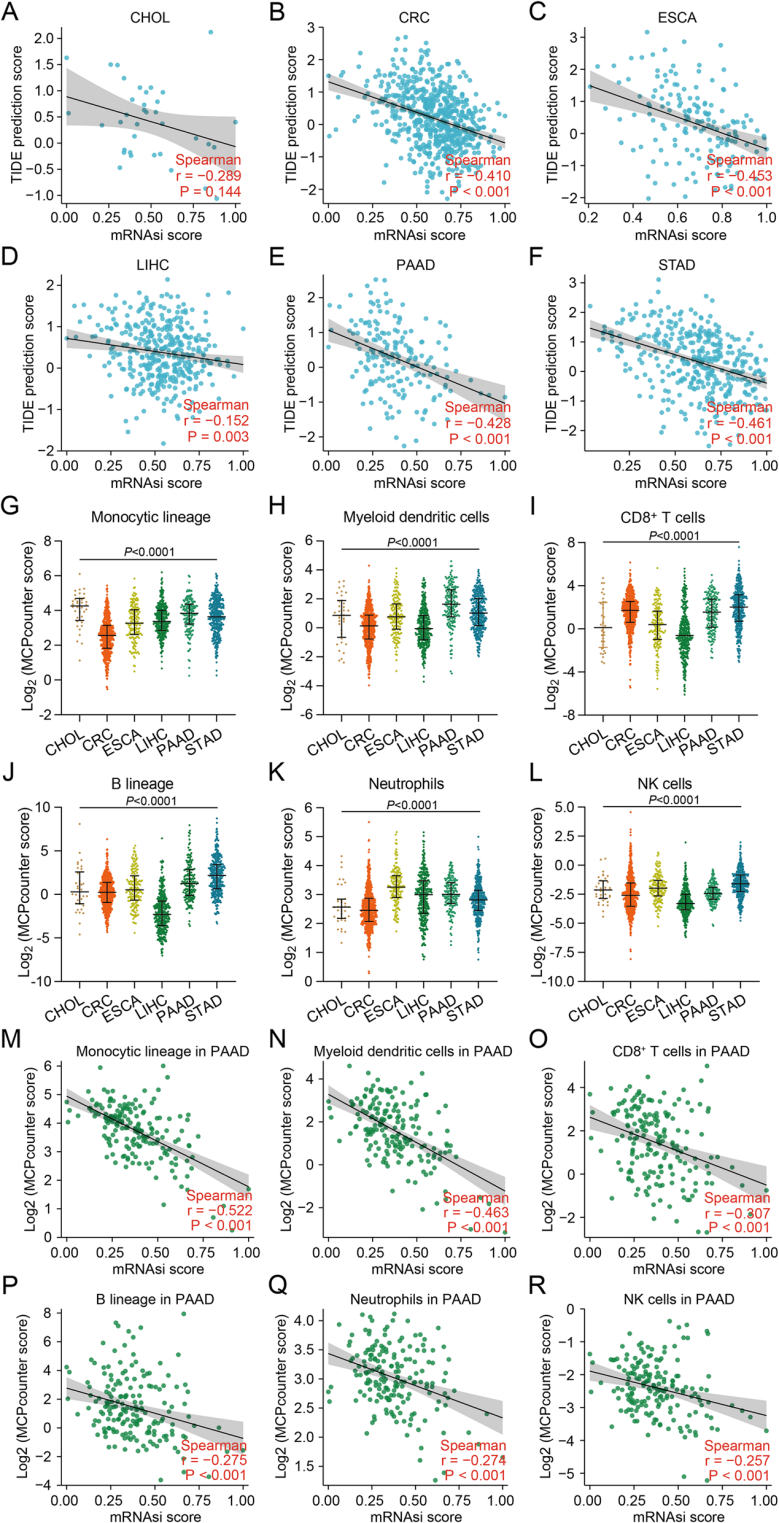
Association of cancer stemness with immunotherapy response across six gastrointestinal cancers. **A-F** Spearman’s correlation of TIDE prediction scores with stemness index (mRNAsi) scores in (**A**) CHOL, (**B**) CRC, (**C**) ESCA, (**D**) LIHC, (**E**) PAAD and (**F**) STAD. **G-H** Infiltration of (**G**) monocytic lineage, (**H**) myeloid dendritic cells, (**I**) CD8^+^ T cells, (**J**) B lineage, (**K**) neutrophils and (**L**) NK cells across six gastrointestinal cancers. **M-R** Spearman correlation of mRNAsi scores in PAAD with infiltration of (**M**) monocytic lineage, (**N**) myeloid dendritic cells, (**O**) CD8^+^ T cells, (**P**) B lineage, (**Q**) neutrophils and (**R**) NK cells

Cancer stemness not only underlies chemotherapy resistance but may also exhibit distinct properties of immune evasion [27]. To investigate the effect of cancer stemness on the efficacy of ICB across six gastrointestinal cancer types, we calculated mRNAsi, a gene expression-based stemness index, to estimate the abundance of cancer stemness [22], and utilized TIDE signatures to predict ICB immunotherapy response [23]. Correlation analysis showed that mRNAsi scores were associated with TIDE prediction scores in CRC, ESCA, PAAD, and STAD (Fig. 1B, C, E, F) instead of LIHC (Fig. 1D), suggesting that cancer stemness indeed contributes to insensitivity to immunotherapy in most gastrointestinal cancer types. While CHOL stemness had little impact on ICB efficacy (Fig. 1A), it cannot be excluded that the low number of CHOL cases could lead to bias and inaccuracy.

The outcome of ICB is linked to the quality and magnitude of tumour-infiltrating immune cells within the tumour microenvironment [28]. To have a comprehensive understanding of immune heterogeneity across six gastrointestinal cancer types, we used MCP-counter, a deconvolution approach, to characterize the cell composition of the immune microenvironment from their gene expression profiles [24]. PAAD and STAD had a higher proportion of monocytic lineage, myeloid dendritic cells, CD8^+^ T cells and B lineage (Fig. 1G-J), which implies that PAAD and STAD suppress anti-cancer immunity in spite that those two cancer types are populated with antigen-presenting cells and cytotoxic cells. On the other hand, the proportion of monocytic lineage and neutrophils in CRC was the lowest among six gastrointestinal cancers (Fig. 1G, K), which reveals that the CRC microenvironment could be characterized by a lack of antigen presentation by the innate immune system. LIHC had low infiltration of lymphoid lineage cells including CD8^+^ T cells, B cells, and natural killer (NK) cells (Fig. 1I, J, L), which can be classified into the immune desert subtype.

To verify whether cancer stemness affects immune infiltrates, we evaluated the association of cancer stemness enrichment with the infiltration of different immunocytes across six gastrointestinal cancer types. The mRNAsi scores of CRC, ESCA, PAAD and STAD were associated with their respective infiltration of myeloid dendritic cells (Fig. 1M-R, Figure S1B, C, E). Besides, the mRNAsi scores of CRC and PAAD also had an association with the infiltration of monocytic lineage, whereas no association of CHOL and LIHC with either myeloid or lymphatic cells was observed (Figure S1A, D). These results suggest that cancer stemness tends to impede the infiltration of the innate immune system, including monocytic lineage and dendritic cells, rather than the adaptive immune system, including CD8^+^ T cells, B cell lineage and neutrophils.

### Identification of a stemness-related inhibitory immune checkpoint in pancreatic cancer

**Fig. 2:**
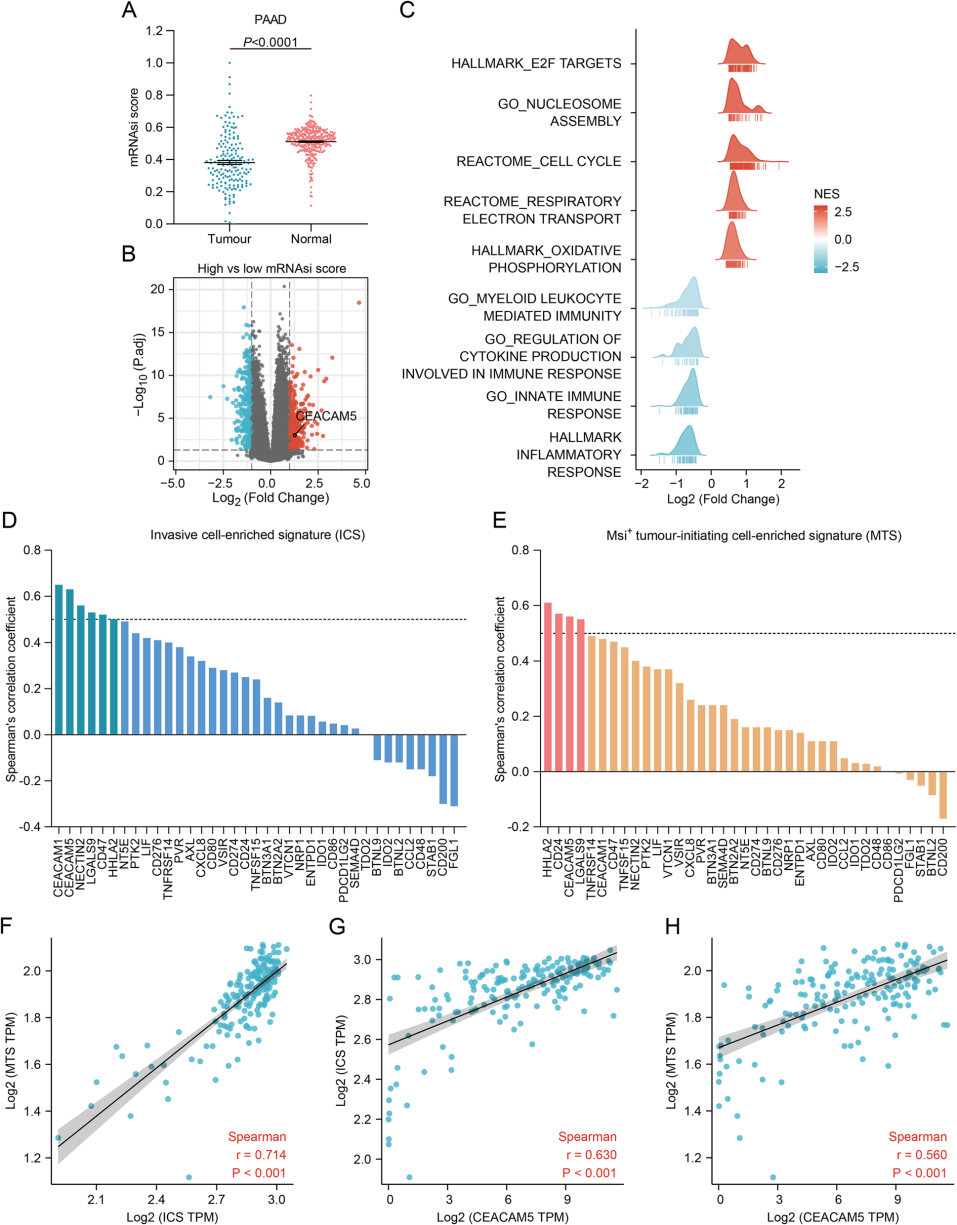
Identification of a stemness-related inhibitory immune checkpoint in pancreatic cancer. **A** Differential levels of mRNAsi scores in pancreatic cancer and paired normal tissues. **B** Volcano plot for differentially expressed genes between pancreatic cancers with high *vs.* low mRNAsi scores. **C** Expression profiles of the inhibitory immune checkpoints in pancreatic cancers with high *vs.* low mRNAsi scores. **D** Ridgeline plot for gene set enrichment analysis of differentially expressed genes between pancreatic cancers with high *vs.* low mRNAsi scores. **E** Spearman's correlation of the inhibitory immune checkpoints with invasive cell-enriched signature (ICS) in pancreatic cancer. The threshold of the correlation coefficient was set at 0.5. **F** Spearman's correlation of the inhibitory immune checkpoints with Msi^+^ tumour-initiating cell-enriched signature (MTS) in pancreatic cancer. The threshold of the correlation coefficient was set at 0.5. **G** Spearman's correlation between ICS and MTS in pancreatic cancer. **H** Spearman’s correlation between CEACAM5 expression and ICS in pancreatic cancer. **I** Spearman's correlation between CEACAM5 expression and MTS in pancreatic cancer

Since cancer stemness can regulate the immune microenvironment, we intended to characterize pancreatic cancer stemness and figure out their way of immune evasion. We found that the mRNAsi scores of tumour tissues were lower than that of normal tissues (Fig. 2A), which means cancer stemness is weaker than normal tissue stemness in the pancreas. Next, we compared the differentially expressed genes between pancreatic cancers with high and low mRNAsi scores to explore the changes in the transcriptomic level. The selection criteria p.adj < 0.05 and |log_2_FC|> 1 resulted in the identification of 289 up-regulated genes and 361 down-regulated genes (Fig. 2B). GSEA showed that the transcriptional signature of pancreatic cancer stemness was correlated with cell cycle-related targets of E2F transcription factors, nucleosome assembly, respiratory electron transport and oxidative phosphorylation (Fig. 2C). This reveals that the maintenance of pancreatic cancer stemness may depend on oxidative phosphorylation instead of glycolysis to produce ATPs more efficiently. It is worth noting that the program of pancreatic cancer stemness was inversely correlated with cytokine production, myeloid leukocyte-mediated immunity, and innate immune response (Fig. 2C). Given that pancreatic cancer stemness negatively regulated anti-cancer immunity, we screened out CEACAM5, which was highly expressed in the high mRNAsi score group, among the 37 selected inhibitory immune checkpoint genes (Fig. 2B). Additionally, we evaluated the correlation of those selected inhibitory immune checkpoints with two individual sets of gene signatures that are representative of pancreatic cancer stem cells. Invasive cell-enriched signature (ICS) is enriched in cancer stem cell-like ductal cells with invasive potential [29]. Msi^+^ tumour-initiating cell-enriched signature (MTS) was generated by comparison of the gene expression profiles of drug-resistant Msi^+^ cancer stem cells and differentiated tumour cells [30]. As expected, ICS and MTS were closely correlated in pancreatic cancer (Fig. 2F), indicating that a high correlation with either of the two individual signatures strongly signifies pancreatic cancer stemness. Correlation analysis showed that the expression of CEACAM1, CEACAM5, NECTIN2, LGALS9, CD47 and HHLA2 was found significantly correlated with ICS, respectively (Fig. 2D, Figure S2A). Meanwhile, the expression of HHLA2, CD24, CEACAM5 and LGALS9 was found significantly correlated with MTS, respectively (Fig. 2E, Figure S2B). Notably, only CEACAM5 expression had a close correlation with both ICS and MTS (Fig. 2G, H). Taken together, CEACAM5 was a potential stemness-related inhibitory immune checkpoint in pancreatic cancer.

**Fig. 3:**
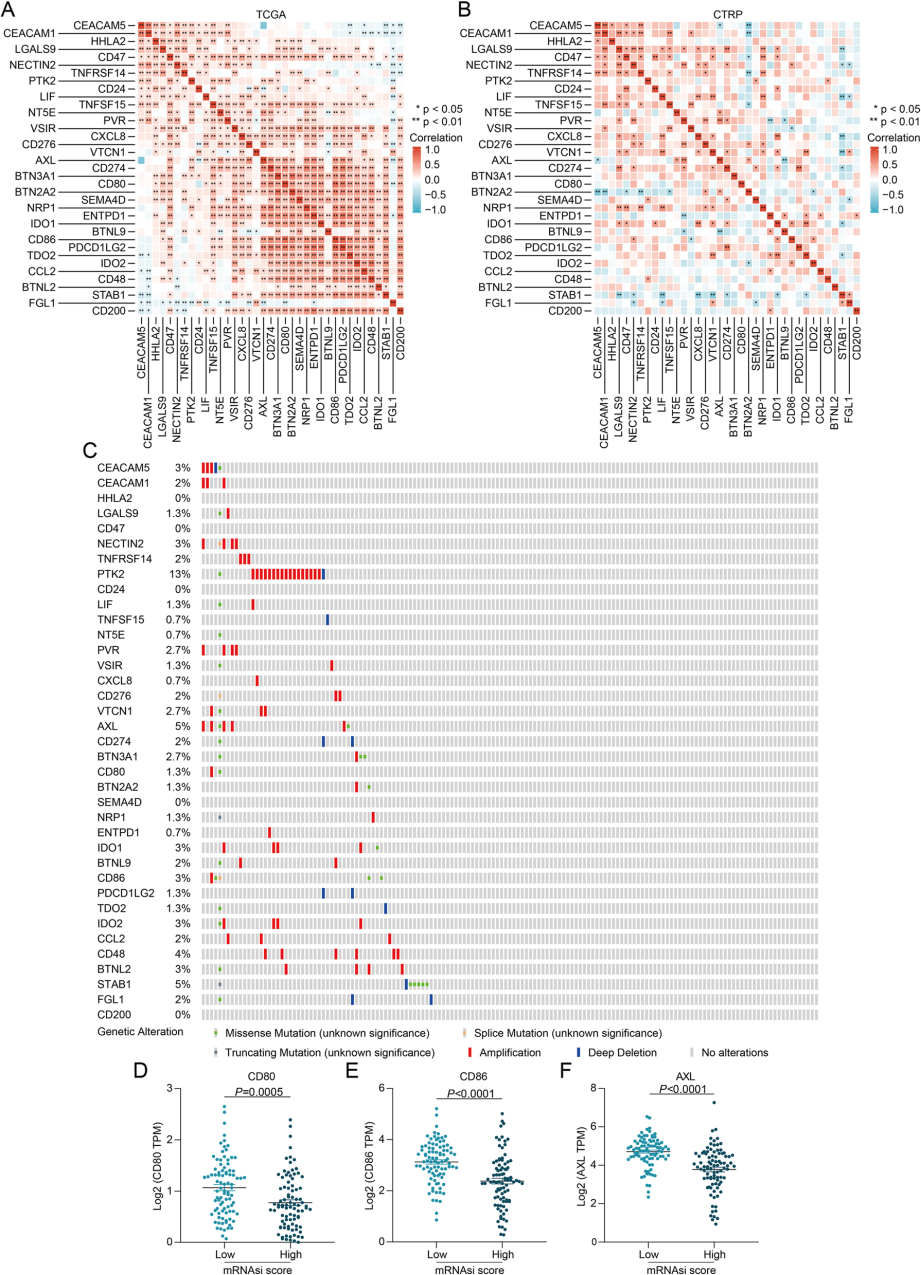
Mutual relevance of the inhibitory immune checkpoints in pancreatic cancer. **A** Profile of Spearman's correlation between the inhibitory immune checkpoints in pancreatic cancer based on the TCGA data. **B** Profile of Pearson’s correlation between the inhibitory immune checkpoints in pancreatic cancer based on the CTRP data. **C** The landscape of genetic alterations of the inhibitory immune checkpoints in pancreatic cancer. **D-F** The levels of (**D**) CD80, (**E**) CD86 and (**F**) AXL expression in pancreatic cancers with high *vs.* low mRNAsi scores

As heterogeneity also exists in cancer stem cells [31], we wondered whether different subpopulations of pancreatic cancer stem cells exhibit distinct inhibitory immune checkpoints except for CEACAM5. When correlation analysis was performed, we adopted tumour purity adjustment to reduce the bias caused by the mixture with immunocytes during data processing. In both TCGA and CTRP data, CEACAM5 was found to have a close correlation with CEACAM1 (Fig. 3A, B). Although CEACAM1 was not proven highly expressed in the high mRNAsi score group, or closely correlated with ICS and MTS (Fig. 2B, E, F), it can be inferred that CEACAM1^+^ CEACAM5^+^ cancer cells may be a small subpopulation of CEACAM5^+^ pancreatic cancer stem cells endowed with an elevated immune privilege. Besides, mutual exclusivity analysis based on mutation and copy number data demonstrated that CEACAM5 exhibited significant co-occurrence with CD80, CD86 and AXL (Fig. 3C, Table 1). Correspondingly, CD80, CD86 and AXL levels were higher in the low mRNAsi score group than in the high mRNAsi score group (Fig. 3D-F). These results imply that CD80, CD86 and AXL may be responsible for protecting pancreatic cancer non-stem cells from immune clearance while CEACAM5 disrupts immune clearance of cancer stem cells.

**Table 1:** Mutual exclusivity analysis of the inhibitory immune checkpoints in pancreatic cancer

A	B	Neither	A Not B	B Not A	Both	Log2 Odds Ratio	*p*-Value	q-Value	Tendency
BTN2A2	BTNL2	144	0	3	2	> 3	< 0.001	0.046	Co-occurrence
CD274	PDCD1LG2	146	1	0	2	> 3	< 0.001	0.03	Co-occurrence
CD274	FGL1	145	1	1	2	> 3	< 0.001	0.046	Co-occurrence
CD276	BTNL9	145	1	1	2	> 3	< 0.001	0.046	Co-occurrence
CD80	CD86	144	0	3	2	> 3	< 0.001	0.046	Co-occurrence
CEACAM5	CD86	142	2	2	3	> 3	< 0.001	0.03	Co-occurrence
CEACAM5	AXL	140	2	4	3	> 3	< 0.001	0.046	Co-occurrence
CEACAM5	CD80	144	3	0	2	> 3	< 0.001	0.046	Co-occurrence
IDO1	IDO2	143	1	1	4	> 3	< 0.001	< 0.001	Co-occurrence
NECTIN2	PVR	144	1	0	4	> 3	< 0.001	< 0.001	Co-occurrence
NECTIN2	AXL	141	1	3	4	> 3	< 0.001	0.002	Co-occurrence
PVR	AXL	141	1	4	3	> 3	< 0.001	0.03	Co-occurrence
VTCN1	CD80	145	2	0	2	> 3	< 0.001	0.046	Co-occurrence

### Function and regulation of the stemness-related inhibitory immune checkpoint in pancreatic cancer

**Fig. 4:**
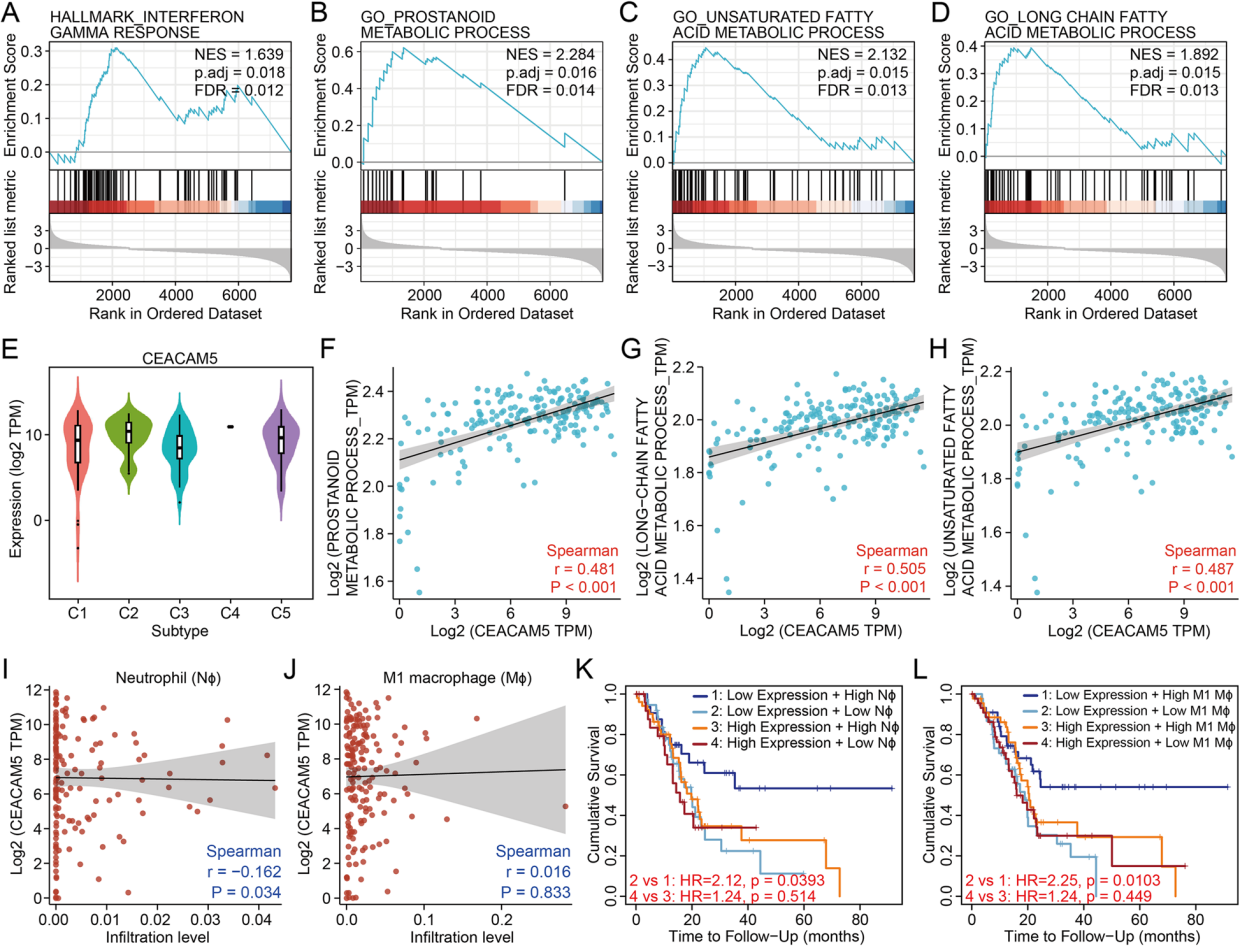
Function and regulation of the stemness-related inhibitory immune checkpoint in pancreatic cancer. **A-D** GSEA of pancreatic cancers with high *vs.* low expression of CEACAM5. **A** Interferon-γ response. **B** Prostanoid metabolic process. **C** Unsaturated fatty acid metabolic process. **D** Long-chain fatty acid metabolic process. **E** Violin plots for CEACAM5 expression in multiple immune subtypes of pancreatic cancer (*P* = 0.0125). C1, wound healing; C2, IFN-γ dominant; C3, inflammatory; C4, lymphocyte depleted; C5, TGF-β dominant. **F** Spearman’s correlation between CEACAM5 expression and prostanoid metabolic process in pancreatic cancer. **G** Spearman’s correlation between CEACAM5 expression and unsaturated fatty acid metabolic process in pancreatic cancer. **H** Spearman’s correlation between CEACAM5 expression and long-chain fatty acid metabolic process in pancreatic cancer. **I** Spearman’s correlation between CEACAM5 expression and infiltration of neutrophils in pancreatic cancer. **J** Spearman’s correlation between CEACAM5 expression and infiltration of M1 macrophages in pancreatic cancer. **K** Kaplan–Meier curves for overall survival split by level of CEACAM5 expression and infiltration level of neutrophils in pancreatic cancer. **L** Kaplan–Meier curves for overall survival split by level of CEACAM5 expression and infiltration level of M1 macrophages in pancreatic cancer

Since the interplay between cancer stemness and the immune microenvironment affects cancer progression [32], we wondered which immune environment CEACAM5^+^ pancreatic cancers with high stemness tend to reside in. The levels of CEACAM5 were found the highest in the IFN-γ dominant pancreatic cancers (Fig. 4E, Figure S5A) which generate the most complex intra-tumour heterogeneity with a high proliferation rate [26]. Despite high infiltration of M1 macrophages and CD8^+^ T cells in the IFN-γ dominant pancreatic cancers [26], poor prognosis reflected the potential inhibitory effect of CEACAM5^+^ cancer cells on anti-cancer immunity in pancreatic cancer. To further clarify the clinical significance of this stemness-related immune checkpoint, we estimated the influence of CEACAM5 expression on immunocyte infiltration and activity by correlation and survival analysis. A more elaborated deconvolution approach CIBERSORT was utilized to distinguish immunocyte composition in the tumour microenvironment [25]. As a result, no association of CEACAM5 expression with infiltration of either innate or adaptive immunocytes was found (Fig. 4I and J, Figure S3A-T). However, any patient group with high levels of CEACAM5 expression had a high risk of poor overall survival, even if accompanied by high infiltration of M1 macrophages or neutrophils (Fig. 4K and L, Figure S4A-T). Consistently, the GEO dataset with accession number GSE21501 also showed that the patients with low levels of CEACAM5 expression and high infiltration of M1 macrophages or neutrophils had the best overall survival (Figure S5C and B). It can be inferred that CEACAM5^+^ pancreatic cancer cells may inhibit the tumoricidal function of M1 macrophages and neutrophils despite no impact on immunocyte infiltration. Thus, stemness-high pancreatic cancers may impair innate immunity such as macrophage through the expression of CEACAM5.

**Fig. 5:**
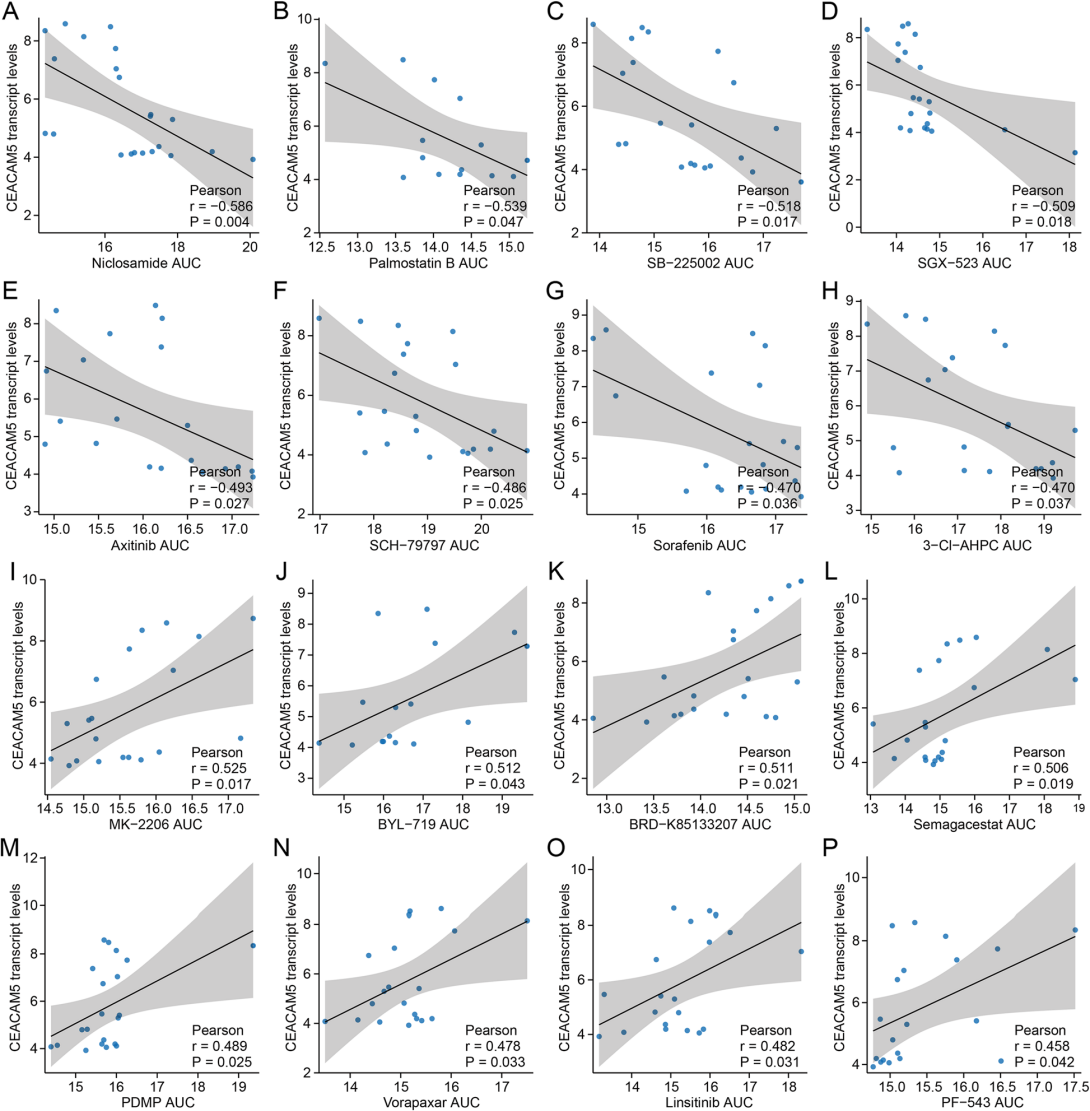
Association of CEACAM5 expression with and sensitivity to small molecular inhibitors. **A-H** Pearson’s correlation between CEACAM5 expression and drug sensitivity to (**A**) Niclosamide, an inhibitor of STAT3 signalling, (**B**) Palmostatin B, an inhibitor of acyl-protein thioesterase 1, (**C**) SB-225002, an inhibitor of chemokine receptor 2, (**D**) SGX-523, an inhibitor of MET, (**E**) Axitinib, an inhibitor of VEGFRs, c-KIT, and PDGFR α and β, (**F**) SCH-79797, an antagonist of proteinase-activated receptor 1, (**G**) Sorafenib, an inhibitor of BRAF, CRAF, and VEGFR2, and (**H**) 3-CL-AHPC, a binder of nuclear receptor SHP. **I-P** Pearson’s correlation between CEACAM5 expression and drug sensitivity to (**I**) MK-2206, an inhibitor of AKT1, (**J**) BYL-719, an inhibitor of PI3K catalytic subunit α, (**K**) BRD-K85133207, an inhibitor of HDAC1, (**L**) Semagacestat, an inhibitor of γ-secretase, (**M**) PDMP, an inhibitor of ceramide glucosyltransferase, (**N**) Vorapaxar, an antagonist of proteinase-activated receptor 1, (**O**) Linsitinib, an inhibitor of insulin-like growth factor 1 receptor and insulin receptor, and (**P**) PF-543, an inhibitor of sphingosine kinase-1

To figure out the potential regulatory pathways, we characterized CEACAM5^high^ pancreatic cancers at the transcriptomic level. GSEA showed that CEACAM5^high^ pancreatic cancers were enriched with pathways for IFN-γ response, prostanoid metabolic process, and long-chain unsaturated fatty acid metabolic process (Fig. 4A-D). Given that IFN-γ switches the status of long-term haematopoietic stem cells from quiescence to proliferation [33], proliferating pancreatic cancers with high stemness may, to some extent, depend on IFN-γ stimulus to sustain cancer progression. As prostaglandin E2 can shape an immunosuppressive barrier in the tumour microenvironment [34], the prostanoid metabolic process is likely to promote the immune resistance of CEACAM5^+^ stemness-high pancreatic cancers. Considering that long-chain unsaturated fatty acid elongation supports membrane architecture and signalling transduction in cancer stem cells [35, 36], the long-chain unsaturated fatty acid metabolic process may modulate the immune privilege of cancer stem cells within stemness-high pancreatic cancers. Meanwhile, those three metabolic processes were found associated with CEACAM5 expression as expected (Fig. 4F-H), suggesting prostanoid and long-chain unsaturated fatty acid metabolic processes could regulate CEACAM5 expression to influence the immune evasion of stemness-high pancreatic cancers. Furthermore, CellMinerCBD was used to screen out the potential drugs sensitive to stemness-high pancreatic cancers with high CEACAM expression. The pancreatic cancer cell lines with higher levels of CEACAM5 generated fewer ATPs when exposed to niclosamide, palmostatin, SB-225002, SGX-523, axitinib, SCH-79797, sorafenib and 3-CI-AHPC (Fig. 5A-H), which means that those inhibitors could efficiently target CEACAM5^high^ pancreatic cancer cells. Conversely, treatment with MK-2206, BYL-719, BRD-K85133207, semagacestat, PDMP, vorapaxar, linsitinib and PF-543 had a better impact on the pancreatic cancer cell lines with lower levels of CEACAM5 (Fig. 5I-P), which means that those inhibitors could be used for clearance of CEACAM5^low/−^ pancreatic cancer cells. Therefore, blockade of IFN-γ signalling or those metabolic processes, and application of those small inhibitors may synergize with anti-CEACAM5 antibodies to improve the elimination of immune evasion in stemness-high pancreatic cancers.

## Discussion

Immunotherapy has to rise to a new level in the context of tumour heterogeneity. As a source of heterogeneity, cancer stem cells have been proposed to be responsible for local recurrence, distant metastasis, and resistance to existing therapies. Precision immunotherapy targeting tumour heterogeneity requires a comprehensive understanding of cancer stem cell immunology. In this study, we found the impact of cancer stemness on immunotherapy responses to pancreatic cancer and identified CEACAM5 as a candidate stemness-related inhibitory immune checkpoint in pancreatic cancer. Levels of CEACAM5 expression were higher in the IFN-γ dominant pancreatic cancer than in the other immune subtypes of pancreatic cancer. The patient group with a high level of CEACAM5 expression and low infiltration of neutrophils or M1 macrophages had poor overall survival. Moreover, prostanoid and long-chain unsaturated fatty acid metabolic processes enriched in stemness-high pancreatic cancers were associated with CEACAM5 expression.

Manipulating the immune system to eliminate the source of tumour heterogeneity remained at the hypothetical stage until the association between cancer stemness and immune evasion was vaguely observed. The inconsistent data on the tumour formation frequency of melanoma-initiating cells in the mouse models with different immunological properties reflected that malignant behaviours of cancer stem cells depend on their immune privilege to some extent [18]. Analysis of gene-expression-based metrics showed the negative association between the presence of a stem cell-like phenotype and anticancer immunity across 21 solid cancers [37], highlighting suppressive signals transduced from cancer stem cells to the immune microenvironment. Previously, we reported CD200 and CD276, respectively, as candidate innate and adaptive immune checkpoints in breast cancer stem cells [38]. Coincidentally, some head and neck squamous cell carcinoma stem cells especially upregulated CD276 to evade host immune responses provoked by CD8^+^ T cells [20]. Another subset of TGF-β-responsive squamous cell carcinoma stem cells acquired CD80 to modulate cytotoxic T cell attack during immunotherapy [19]. Although breast cancer and head and neck squamous cell carcinoma are ectodermal tumours which pancreatic cancer cannot be classified into, the similar immune privilege of cancer stem cells may still exist in pancreatic cancer. Following the principle, our study revealed CEACAM5 as a candidate stemness-related inhibitory immune checkpoint in pancreatic cancer. Recently, it has been demonstrated that CEACAM5 overexpression is a reliable characteristic of CD133-positive colorectal cancer stem cells [39]. Meanwhile, in patients with metastatic pancreatic cancer, median overall survival became shorter as the serum CEACAM5 levels increased [40]. Elevated serum CEACAM5 levels could also predict early recurrence after curative resection of PDAC [41]. It is reasonable to infer that proliferating CEACAM5^+^ pancreatic cancer stem cells may act as deadly seeds in recurrence and that detection of those cells could be an approach to determining metastatic burden. Therefore, uncovering stemness-high pancreatic cancers with high CEACAM5 expression increase the possibility of applying immunotherapy for the clearance of pancreatic cancer stem cells.

The heterophilic interaction between CEACAM5 on tumour cells and CEACAM1 on NK cells has been demonstrated to inhibit NK cell-mediated anti-cancer immunity [42]. The binding of CEACAM5 to CD8α and CD1d facilitated CD8^+^ T cells to acquire suppressive functions and then reduced the proliferation of CD4^+^ T cells [43]. Our study indicated that the infiltration of M1 macrophages and neutrophils in pancreatic cancer can signify prolonged patient survival and that CEACAM5 in stemness-high pancreatic cancers could impair the tumoricidal function of M1 macrophages and neutrophils. The discovery of the inhibitory role of CEACAM5^+^ tumour cells in the innate immune cells increases emphasis on harnessing innate immunity in immunotherapy, particularly when most of the current immunomodulatory approaches have focused on unleashing effector T cells. Despite good prognosis predicted by T-cell infiltration in the tumour microenvironment [44] and the initial success of chimeric antigen receptor (CAR) T-cell therapy in some haematological malignancies [45], the onset and maintenance of T-cell responses and the development of long-term memory T cells depend on the activation of the innate immune system. In the preclinical study, genetic ablation or antibody blockade of CD24 resulted in a macrophage-dependent reduction of ovarian and breast cancer growth [46]. In acute myeloid leukaemia, VCAM1 inhibition or deletion reduced tumour burden and extends survival through restoring clearance by mononuclear phagocytes [47]. Therefore, enhancing the effector responses of innate immunity, such as phagocytosis for macrophages and polymorphonuclear cells, should be the main goal of immunotherapies targeting pancreatic cancer stem cells.

Compared with differentiated bulk tumour cells, cancer stem cells reside in a distinct niche which supports their contribution to recurrence and metastasis. Our study revealed that long-chain unsaturated fatty acids may regulate CEACAM5 expression and that proliferating stemness-high pancreatic cancers with high CEACAM5 expression may adopt a dual phenotype of oxidative phosphorylation and fatty acid oxidation. Previously, increased serum levels of fatty acid synthase, a metabolic enzyme that catalyses the synthesis of long-chain fatty acids, were observed in pancreatic cancer patients [48], and accumulation of long-chain fatty acids in the tumour microenvironment drove dysfunction in intra-pancreatic CD8^+^ T cells [49]. Beyond that, genetic attenuation or pharmacological inhibition of the metabolic process of long-chain fatty acids impaired mitochondrial respiration and fatty acid oxidation triggered decreased proliferation and tumour cell engraftment [50]. It can be inferred that the metabolism of long-chain unsaturated fatty acids may orchestrate immune evasion of pancreatic cancer stem cells by regulating inhibitory immune checkpoints. Furthermore, our study confirmed the association of prostanoid metabolism with CEACAM5 expression. Consistently, levels of prostaglandin E2 (PGE2), one of the members of the prostanoid, correlated with cancer stem cell markers in colorectal cancer, including CD133, CD44, LRG5, and SOX2 [51]. Mesenchymal stem cell-secreted PGE2 promoted the expansion of colorectal cancer stem cells and the formation of hepatic metastases, which administration of celecoxib, an inhibitor of prostaglandin-endoperoxide synthase 2, prominently restrained [51, 52]. Mechanically, PGE2 acted in a paracrine manner to activate Akt/GSK-3/β-catenin signalling, thus inducing an increase in ALDH^high^ cancer stem cell-enriched population [52, 53]. Thus, prostanoid metabolism may be another process involved in the regulation of inhibitory immune checkpoints to sustain pancreatic cancer stem cells in the immune microenvironment. Taken together, inhibition of the metabolic process of long-chain unsaturated fatty acid and prostanoid could synergize with immune checkpoint blockade to break down the immune privilege of cancer stem cells within stemness-high pancreatic cancers.

The current study still has several limitations that need to be addressed in the future. First of all, our findings were based on bioinformatic analysis of bulk transcriptomic sequencing data. A landscape of pancreatic cancer cells at single-cell levels will provide much more valuable insights into the immune privilege of pancreatic cancer stem cells. Secondly, our results need to be verified by further experiments in patient-derived xenografts and spontaneous tumour models, such as gene knockout, limiting dilution assay and lineage tracing. Thirdly, the synergistically therapeutic effect of a combination of ICB and relevant metabolism inhibition on local recurrence and distant metastasis caused by pancreatic cancer stem cells requires clinical trials to manifest. Despite those limitations, the identification of stemness-related inhibitory immune checkpoints and relevant regulatory metabolisms is anticipated to promote the development of cancer stem cell immunology.

## Conclusions

Our study reveals CEACAM5 as a stemness-related inhibitory immune checkpoint in pancreatic cancer. Synergistic inhibition of stemness-related regulatory metabolisms, including prostanoid and long-chain unsaturated fatty acid metabolic processes, may improve the efficacy of ICB treatment which is aimed at eliminating immune evasion in stemness-high pancreatic cancers with high CEACAM5 expression. The combination treatment will provide a novel and efficient strategy for precision immunotherapy targeting tumour heterogeneity.

## Supplementary Information

**Additional file 1: Fig. S1.** Association of cancer stemness with immune infiltrates in five other gastrointestinal cancers. (A) Spearman correlation of mRNAsi scores in CHOL with infiltration of monocytic lineage, myeloid dendritic cells, CD8^+^ T cells, B lineage, neutrophils and NK cells. (B) Spearman correlation of mRNAsi scores in CRC with infiltration of monocytic lineage, myeloid dendritic cells, CD8^+^ T cells, B lineage, neutrophils and NK cells. (C) Spearman correlation of mRNAsi scores in ESCA with infiltration of monocytic lineage, myeloid dendritic cells, CD8^+^ T cells, B lineage, neutrophils and NK cells. (D) Spearman correlation of mRNAsi scores in LIHC with infiltration of monocytic lineage, myeloid dendritic cells, CD8^+^ T cells, B lineage, neutrophils and NK cells. (E) Spearman correlation of mRNAsi scores in STAD with infiltration of monocytic lineage, myeloid dendritic cells, CD8^+^ T cells, B lineage, neutrophils and NK cells.

**Additional file 2: Fig. S2.** Statistical significance of association between the inhibitory immune checkpoints and two individual sets of gene signatures in pancreatic cancer. (A) The p-value of Spearman’s correlation between the inhibitory immune checkpoints and ICS. (B) The p-value of Spearman’s correlation between the inhibitory immune checkpoints and MTS.

**Additional file 3: Fig. S3.** Association of CEACAM5 expression with immune infiltrates in pancreatic cancer. (A-J) Spearman’s correlation of CEACAM5 expression with infiltration of (A) CD8^+^ T cells, (B) naïve CD4^+^ T cells, (C) activated memory CD4^+^ T cells, (D) resting memory CD4^+^ T cells, (E) Tregs, (F) γδ T cells, (G) T follicular helper cells, (H) naïve B cells, (I) memory B cells, (J) plasma cells. (K-T) Spearman’s correlation of CEACAM5 expression with infiltration of (K) monocytes, (L) M0 macrophages, (M) M2 macrophages, (N) activated myeloid dendritic cells, (O) resting myeloid dendritic cells, (P) activated NK cells, (Q) resting NK cells, (R) eosinophils, (S) activated mast cells, (T) activated mast cells.

**Additional file 4: Fig. S4.** Prognostic value of CEACAM expression plus immune infiltration level in patients with pancreatic cancer. (A-J) Kaplan-Meier curves for overall survival split by level of CEACAM5 expression and infiltration level of (A) CD8^+^ T cells, (B) naïve CD4^+^ T cells, (C) activated memory CD4^+^ T cells, (D) resting memory CD4^+^ T cells, (E) Tregs, (F) γδ T cells, (G) T follicular helper cells, (H) naïve B cells, (I) memory B cells, (J) plasma cells. (K-T) Kaplan-Meier curves for overall survival split by level of CEACAM5 expression and infiltration level of (K) monocytes, (L) M0 macrophages, (M) M2 macrophages, (N) activated myeloid dendritic cells, (O) resting myeloid dendritic cells, (P) activated NK cells, (Q) resting NK cells, (R) eosinophils, (S) activated mast cells, (T) activated mast cells.

**Additional file 5: Fig. S5.** Validation using the GSE21501 dataset. (A) Violin plots for CEACAM5 expression in multiple immune subtypes of pancreatic cancer (*P*=0.0022). C1, wound healing; C2, IFN-γ dominant; C3, inflammatory; C4, lymphocyte depleted; C5, TGF-β dominant. (B) Kaplan-Meier curves for overall survival split by level of CEACAM5 expression and infiltration level of neutrophils in pancreatic cancer. (C) Kaplan-Meier curves for overall survival split by level of CEACAM5 expression and infiltration level of M1 macrophages in pancreatic cancer.

## Data Availability

The datasets analyzed during the current study are available in the TCGA, CTRP and GSE21501 repository. TCGA: https://portal.gdc.cancer.gov/. CTRP: https://portals.broadinstitute.org/ctrp/. GSE21501: https://www.ncbi.nlm.nih.gov/geo/query/acc.cgi?acc=GSE21501.

## Abbreviations

TCGA: The Cancer Genome Atlas
CTRP: Cancer Therapeutics Response Portal
ICB: Immune checkpoint blockade
CHOL: Cholangiocarcinoma
CRC: Colorectal adenocarcinoma
ESCA: Oesophagal carcinoma
LIHC: Liver hepatocellular carcinoma
PAAD: Pancreatic adenocarcinoma
STAD: Stomach adenocarcinoma
TIDE: Tumour Immune Dysfunction and Exclusion
IFN-γ: Interferon-γ
TGF-β: Transforming growth factor-β
GSEA: Gene set enrichment analysis
ICS: Invasive cell-enriched signature
MTS: Msi^+^ tumour-initiating cell-enriched signature

## References

[1] Sung H, Ferlay J, Siegel RL, Laversanne M, Soerjomataram I, Jemal A, Bray F (2021). Global Cancer Statistics 2020: GLOBOCAN estimates of incidence and mortality worldwide for 36 cancers in 185 countries. CA Cancer J Clin.

[2] Strobel O, Neoptolemos J, Jager D, Buchler MW (2019). Optimizing the outcomes of pancreatic cancer surgery. Nat Rev Clin Oncol.

[3] Park W, Chawla A, O'Reilly EM (2021). Pancreatic cancer: a review. JAMA.

[4] Carlino MS, Larkin J, Long GV (2021). Immune checkpoint inhibitors in melanoma. Lancet.

[5] Wang M, Herbst RS, Boshoff C (2021). Toward personalized treatment approaches for non-small-cell lung cancer. Nat Med.

[6] Royal RE, Levy C, Turner K, Mathur A, Hughes M, Kammula US, Sherry RM, Topalian SL, Yang JC, Lowy I (2010). Phase 2 trial of single agent Ipilimumab (anti-CTLA-4) for locally advanced or metastatic pancreatic adenocarcinoma. J Immunother.

[7] Brahmer JR, Tykodi SS, Chow LQ, Hwu WJ, Topalian SL, Hwu P, Drake CG, Camacho LH, Kauh J, Odunsi K (2012). Safety and activity of anti-PD-L1 antibody in patients with advanced cancer. N Engl J Med.

[8] Bockorny B, Semenisty V, Macarulla T, Borazanci E, Wolpin BM, Stemmer SM, Golan T, Geva R, Borad MJ, Pedersen KS (2020). BL-8040, a CXCR4 antagonist, in combination with pembrolizumab and chemotherapy for pancreatic cancer: the COMBAT trial. Nat Med.

[9] O'Hara MH, O'Reilly EM, Varadhachary G, Wolff RA, Wainberg ZA, Ko AH, Fisher G, Rahma O, Lyman JP, Cabanski CR (2021). CD40 agonistic monoclonal antibody APX005M (sotigalimab) and chemotherapy, with or without nivolumab, for the treatment of metastatic pancreatic adenocarcinoma: an open-label, multicentre, phase 1b study. Lancet Oncol.

[10] Humphris JL, Patch AM, Nones K, Bailey PJ, Johns AL, McKay S, Chang DK, Miller DK, Pajic M, Kassahn KS (2017). Hypermutation in pancreatic cancer. Gastroenterology.

[11] Jiang H, Hegde S, Knolhoff BL, Zhu Y, Herndon JM, Meyer MA, Nywening TM, Hawkins WG, Shapiro IM, Weaver DT (2016). Targeting focal adhesion kinase renders pancreatic cancers responsive to checkpoint immunotherapy. Nat Med.

[12] Wolf Y, Bartok O, Patkar S, Eli GB, Cohen S, Litchfield K, Levy R, Jimenez-Sanchez A, Trabish S, Lee JS (2019). UVB-Induced tumor heterogeneity diminishes immune response in melanoma. Cell.

[13] Nassar D, Blanpain C (2016). Cancer stem cells: basic concepts and therapeutic implications. Annu Rev Pathol.

[14] Massague J, Obenauf AC (2016). Metastatic colonization by circulating tumour cells. Nature.

[15] Hermann PC, Huber SL, Herrler T, Aicher A, Ellwart JW, Guba M, Bruns CJ, Heeschen C (2007). Distinct populations of cancer stem cells determine tumor growth and metastatic activity in human pancreatic cancer. Cell Stem Cell.

[16] Seguin L, Kato S, Franovic A, Camargo MF, Lesperance J, Elliott KC, Yebra M, Mielgo A, Lowy AM, Husain H (2014). An integrin beta(3)-KRAS-RalB complex drives tumour stemness and resistance to EGFR inhibition. Nat Cell Biol.

[17] Wang VM, Ferreira RMM, Almagro J, Evan T, Legrave N, Zaw Thin M, Frith D, Carvalho J, Barry DJ, Snijders AP (2019). CD9 identifies pancreatic cancer stem cells and modulates glutamine metabolism to fuel tumour growth. Nat Cell Biol.

[18] Quintana E, Shackleton M, Sabel MS, Fullen DR, Johnson TM, Morrison SJ (2008). Efficient tumour formation by single human melanoma cells. Nature.

[19] Miao Y, Yang H, Levorse J, Yuan S, Polak L, Sribour M, Singh B, Rosenblum MD, Fuchs E (2019). Adaptive immune resistance emerges from tumor-initiating stem cells. Cell.

[20] Wang C, Li Y, Jia L, Kim JK, Li J, Deng P, Zhang W, Krebsbach PH, Wang CY (2021). CD276 expression enables squamous cell carcinoma stem cells to evade immune surveillance. Cell Stem Cell.

[21] Stratford JK, Bentrem DJ, Anderson JM, Fan C, Volmar KA, Marron JS, Routh ED, Caskey LS, Samuel JC, Der CJ (2010). A six-gene signature predicts survival of patients with localized pancreatic ductal adenocarcinoma. PLoS Med.

[22] Malta TM, Sokolov A, Gentles AJ, Burzykowski T, Poisson L, Weinstein JN, Kaminska B, Huelsken J, Omberg L, Gevaert O (2018). Machine learning identifies stemness features associated with oncogenic dedifferentiation. Cell.

[23] Jiang P, Gu S, Pan D, Fu J, Sahu A, Hu X, Li Z, Traugh N, Bu X, Li B (2018). Signatures of T cell dysfunction and exclusion predict cancer immunotherapy response. Nat Med.

[24] Becht E, Giraldo NA, Lacroix L, Buttard B, Elarouci N, Petitprez F, Selves J, Laurent-Puig P, Sautes-Fridman C, Fridman WH (2016). Estimating the population abundance of tissue-infiltrating immune and stromal cell populations using gene expression. Genome Biol.

[25] Newman AM, Liu CL, Green MR, Gentles AJ, Feng W, Xu Y, Hoang CD, Diehn M, Alizadeh AA (2015). Robust enumeration of cell subsets from tissue expression profiles. Nat Methods.

[26] Thorsson V, Gibbs DL, Brown SD, Wolf D, Bortone DS, Ou Yang TH, Porta-Pardo E, Gao GF, Plaisier CL, Eddy JA (2019). The immune landscape of cancer. Immunity.

[27] Perry JM, Tao F, Roy A, Lin T, He XC, Chen S, Lu X, Nemechek J, Ruan L, Yu X (2020). Overcoming Wnt-beta-catenin dependent anticancer therapy resistance in leukaemia stem cells. Nat Cell Biol.

[28] Paijens ST, Vledder A, de Bruyn M, Nijman HW (2021). Tumor-infiltrating lymphocytes in the immunotherapy era. Cell Mol Immunol.

[29] Ren X, Zhou C, Lu Y, Ma F, Fan Y, Wang C (2021). Single-cell RNA-seq reveals invasive trajectory and determines cancer stem cell-related prognostic genes in pancreatic cancer. Bioengineered.

[30] Lytle NK, Ferguson LP, Rajbhandari N, Gilroy K, Fox RG, Deshpande A, Schurch CM, Hamilton M, Robertson N, Lin W (2019). A multiscale map of the stem cell state in pancreatic adenocarcinoma. Cell.

[31] Zheng H, Pomyen Y, Hernandez MO, Li C, Livak F, Tang W, Dang H, Greten TF, Davis JL, Zhao Y (2018). Single-cell analysis reveals cancer stem cell heterogeneity in hepatocellular carcinoma. Hepatology.

[32] Clara JA, Monge C, Yang Y, Takebe N (2020). Targeting signalling pathways and the immune microenvironment of cancer stem cells - a clinical update. Nat Rev Clin Oncol.

[33] Baldridge MT, King KY, Boles NC, Weksberg DC, Goodell MA (2010). Quiescent haematopoietic stem cells are activated by IFN-gamma in response to chronic infection. Nature.

[34] He X, Smith SE, Chen S, Li H, Wu D, Meneses-Giles PI, Wang Y, Hembree M, Yi K, Zhao X (2021). Tumor-initiating stem cell shapes its microenvironment into an immunosuppressive barrier and pro-tumorigenic niche. Cell Rep.

[35] Gimple RC, Kidwell RL, Kim LJY, Sun T, Gromovsky AD, Wu Q, Wolf M, Lv D, Bhargava S, Jiang L (2019). Glioma stem cell-specific superenhancer promotes polyunsaturated fatty-acid synthesis to support EGFR signaling. Cancer Discov.

[36] Di Carlo C, Sousa BC, Manfredi M, Brandi J, Dalla Pozza E, Marengo E, Palmieri M, Dando I, Wakelam MJO, Lopez-Clavijo AF (2021). Integrated lipidomics and proteomics reveal cardiolipin alterations, upregulation of HADHA and long chain fatty acids in pancreatic cancer stem cells. Sci Rep.

[37] Miranda A, Hamilton PT, Zhang AW, Pattnaik S, Becht E, Mezheyeuski A, Bruun J, Micke P, de Reynies A, Nelson BH (2019). Cancer stemness, intratumoral heterogeneity, and immune response across cancers. Proc Natl Acad Sci U S A.

[38] Shi H, Yang Y (2021). Identification of inhibitory immune checkpoints and relevant regulatory pathways in breast cancer stem cells. Cancer Med.

[39] Gisina A, Novikova S, Kim Y, Sidorov D, Bykasov S, Volchenko N, Kaprin A, Zgoda V, Yarygin K, Lupatov A (2021). CEACAM5 overexpression is a reliable characteristic of CD133-positive colorectal cancer stem cells. Cancer Biomark.

[40] Imaoka H, Mizuno N, Hara K, Hijioka S, Tajika M, Tanaka T, Ishihara M, Hirayama Y, Hieda N, Yoshida T (2016). Prognostic impact of carcinoembryonic antigen (CEA) on patients with metastatic pancreatic cancer: a retrospective cohort study. Pancreatology.

[41] Suzuki S, Shimoda M, Shimazaki J, Maruyama T, Oshiro Y, Nishida K, Sahara Y, Nagakawa Y, Tsuchida A (2018). Predictive early recurrence factors of preoperative clinicophysiological findings in pancreatic cancer. Eur Surg Res.

[42] Stern N, Markel G, Arnon TI, Gruda R, Wong H, Gray-Owen SD, Mandelboim O (2005). Carcinoembryonic antigen (CEA) inhibits NK killing via interaction with CEA-related cell adhesion molecule 1. J Immunol.

[43] Roda G, Jianyu X, Park MS, DeMarte L, Hovhannisyan Z, Couri R, Stanners CP, Yeretssian G, Mayer L (2014). Characterizing CEACAM5 interaction with CD8alpha and CD1d in intestinal homeostasis. Mucosal Immunol.

[44] Pages F, Mlecnik B, Marliot F, Bindea G, Ou FS, Bifulco C, Lugli A, Zlobec I, Rau TT, Berger MD (2018). International validation of the consensus Immunoscore for the classification of colon cancer: a prognostic and accuracy study. Lancet.

[45] June CH, O'Connor RS, Kawalekar OU, Ghassemi S, Milone MC (2018). CAR T cell immunotherapy for human cancer. Science.

[46] Barkal AA, Brewer RE, Markovic M, Kowarsky M, Barkal SA, Zaro BW, Krishnan V, Hatakeyama J, Dorigo O, Barkal LJ (2019). CD24 signalling through macrophage Siglec-10 is a target for cancer immunotherapy. Nature.

[47] Pinho S, Wei Q, Maryanovich M, Zhang D, Balandran JC, Pierce H, Nakahara F, Di Staulo A, Bartholdy BA, Xu J (2022). VCAM1 confers innate immune tolerance on haematopoietic and leukaemic stem cells. Nat Cell Biol.

[48] Walter K, Hong SM, Nyhan S, Canto M, Fedarko N, Klein A, Griffith M, Omura N, Medghalchi S, Kuhajda F (2009). Serum fatty acid synthase as a marker of pancreatic neoplasia. Cancer Epidemiol Biomarkers Prev.

[49] Manzo T, Prentice BM, Anderson KG, Raman A, Schalck A, Codreanu GS, Nava Lauson CB, Tiberti S, Raimondi A, Jones MA (2020). Accumulation of long-chain fatty acids in the tumor microenvironment drives dysfunction in intrapancreatic CD8+ T cells. J Exp Med.

[50] Tcheng M, Roma A, Ahmed N, Smith RW, Jayanth P, Minden MD, Schimmer AD, Hess DA, Hope K, Rea KA (2021). Very long chain fatty acid metabolism is required in acute myeloid leukemia. Blood.

[51] Wang D, Fu L, Sun H, Guo L, DuBois RN (2015). Prostaglandin E2 promotes colorectal cancer stem cell expansion and metastasis in mice. Gastroenterology.

[52] Li HJ, Reinhardt F, Herschman HR, Weinberg RA (2012). Cancer-stimulated mesenchymal stem cells create a carcinoma stem cell niche via prostaglandin E2 signaling. Cancer Discov.

[53] Oshima H, Matsunaga A, Fujimura T, Tsukamoto T, Taketo MM, Oshima M (2006). Carcinogenesis in mouse stomach by simultaneous activation of the Wnt signaling and prostaglandin E2 pathway. Gastroenterology.

