# Supplementary material for: Identification of CEACAM5 as a stemness-related inhibitory immune checkpoint in pancreatic cancer
Source: BMC Cancer. 2022 Dec 9;22:1291. doi: 10.1186/s12885-022-10397-7 (PMC9733357; doi:10.1186/s12885-022-10397-7)
Supplement: Supplementary file 1 — Additional file 1: Fig. S1. Association of cancer stemness with immune infiltrates in five other gastrointestinal cancers. (A) Spearman correlation of mRNAsi scores in CHOL with infiltration of monocytic lineage, myeloid dendritic cells, CD8+ T cells, B lineage, neutrophils and NK cells. (B) Spearman correlation of mRNAsi scores in CRC with infiltration of monocytic lineage, myeloid dendritic cells, CD8+ T cells, B lineage, neutrophils and NK cells. (C) Spearman correlation of mRNAsi scores in ESCA with infiltration of monocytic lineage, myeloid dendritic cells, CD8+ T cells, B lineage, neutrophils and NK cells. (D) Spearman correlation of mRNAsi scores in LIHC with infiltration of monocytic lineage, myeloid dendritic cells, CD8+ T cells, B lineage, neutrophils and NK cells. (E) Spearman correlation of mRNAsi scores in STAD with infiltration of monocytic lineage, myeloid dendritic cells, CD8+ T cells, B lineage, neutrophils and NK cells. [file 12885_2022_10397_MOESM1_ESM.pdf]

Additional file 1

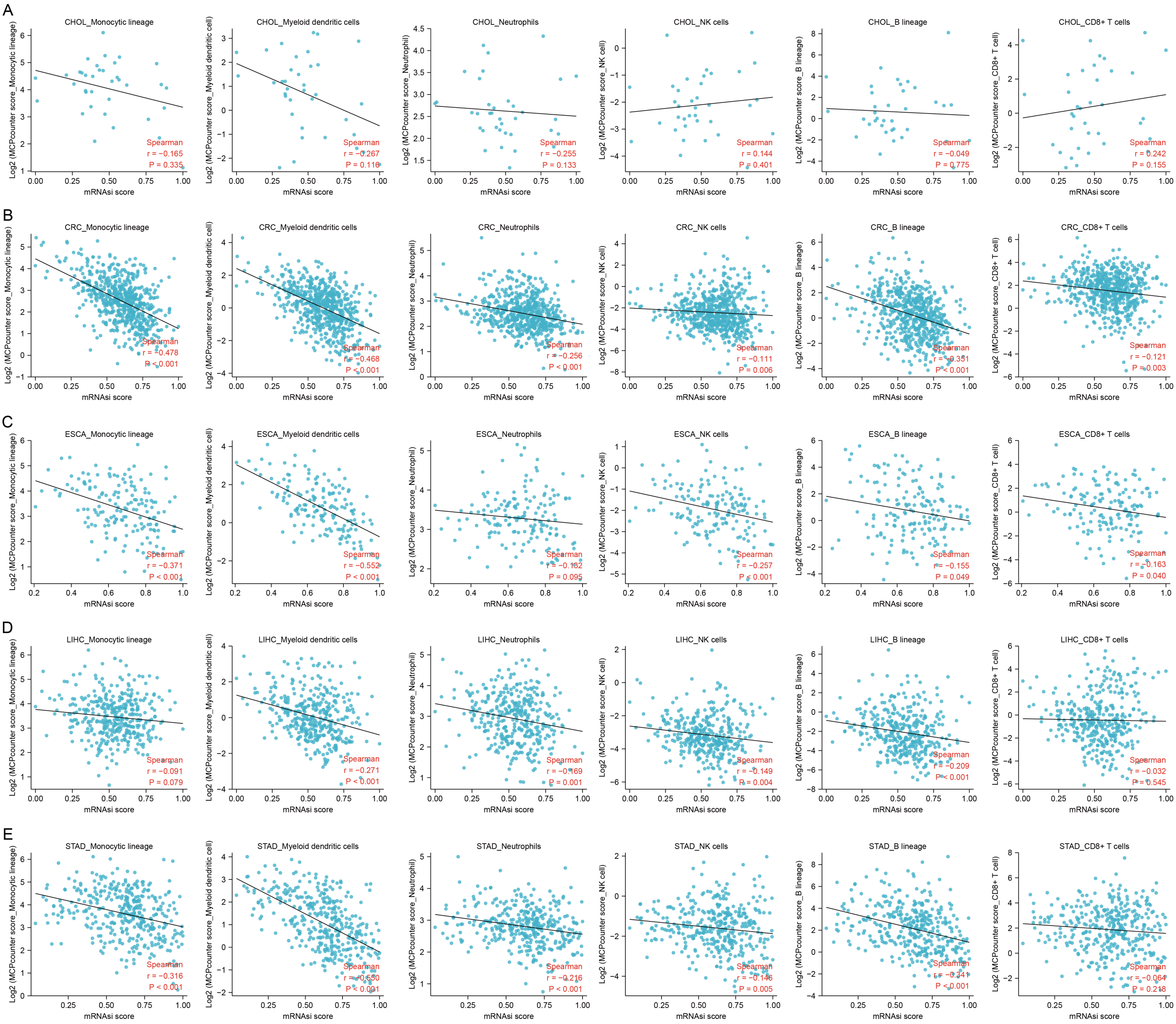

Fig. S1 Association of cancer stemness with immune infiltrates in five other gastrointestinal cancers. (**A**) Spearman correlation of mRNAsi scores in CHOL with infiltration of monocytic lineage, myeloid dendritic cells, $CD8^+$ T cells, B lineage, neutrophils and NK cells. (**B**) Spearman correlation of mRNAsi scores in CRC with infiltration of monocytic lineage, myeloid dendritic cells, $CD8^+$ T cells, B lineage, neutrophils and NK cells. (**C**) Spearman correlation of mRNAsi scores in ESCA with infiltration of monocytic lineage, myeloid dendritic cells, $CD8^+$ T cells, B lineage, neutrophils and NK cells. (**D**) Spearman correlation of mRNAsi scores in LIHC with infiltration of monocytic lineage, myeloid dendritic cells, $CD8^+$ T cells, B lineage, neutrophils and NK cells. (**E**) Spearman correlation of mRNAsi scores in STAD with infiltration of monocytic lineage, myeloid dendritic cells, $CD8^+$ T cells, B lineage, neutrophils and NK cells.
