# Supplementary material for: Identification of CEACAM5 as a stemness-related inhibitory immune checkpoint in pancreatic cancer
Source: BMC Cancer. 2022 Dec 9;22:1291. doi: 10.1186/s12885-022-10397-7 (PMC9733357; doi:10.1186/s12885-022-10397-7)
Supplement: Supplementary file 2 — Additional file 2: Fig. S2. Statistical significance of association between the inhibitory immune checkpoints and two individual sets of gene signatures in pancreatic cancer. (A) The p-value of Spearman’s correlation between the inhibitory immune checkpoints and ICS. (B) The p-value of Spearman’s correlation between the inhibitory immune checkpoints and MTS. [file 12885_2022_10397_MOESM2_ESM.pdf]

Additional file 2

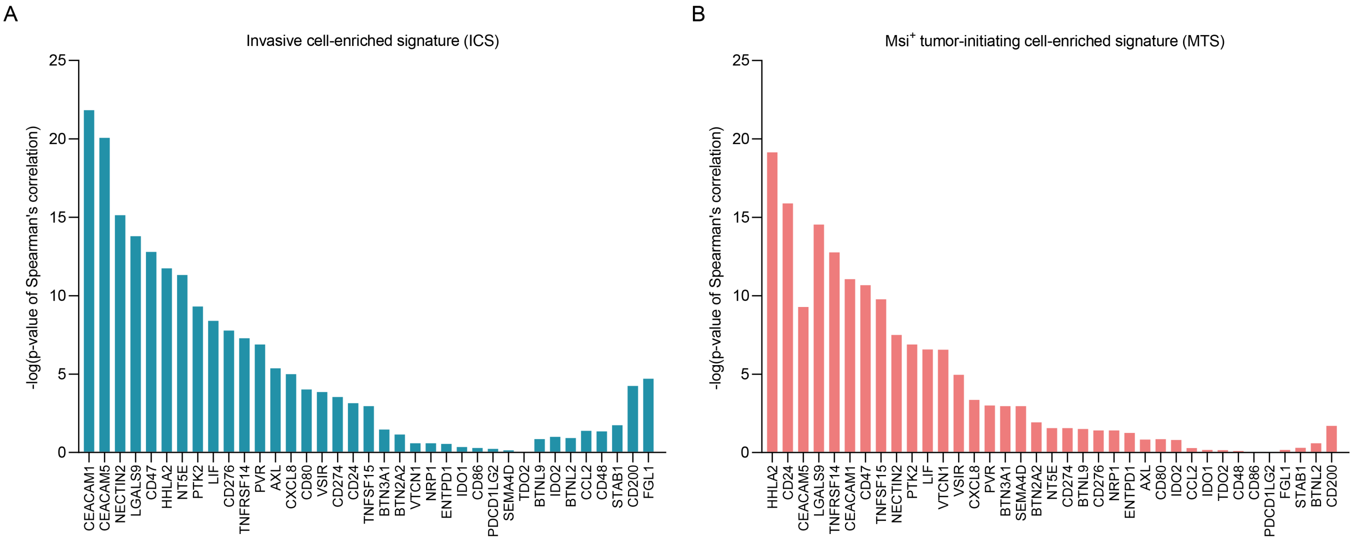

Fig. S2 Statistical significance of association between the inhibitory immune checkpoints and two individual sets of gene signatures in pancreatic cancer. (**A**) The p-value of Spearman's correlation between the inhibitory immune checkpoints and ICS. (**B**) The p-value of Spearman's correlation between the inhibitory immune checkpoints and MTS.
