# Supplementary material for: Identification of CEACAM5 as a stemness-related inhibitory immune checkpoint in pancreatic cancer
Source: BMC Cancer. 2022 Dec 9;22:1291. doi: 10.1186/s12885-022-10397-7 (PMC9733357; doi:10.1186/s12885-022-10397-7)
Supplement: Supplementary file 3 — Additional file 3: Fig. S3. Association of CEACAM5 expression with immune infiltrates in pancreatic cancer. (A-J) Spearman’s correlation of CEACAM5 expression with infiltration of (A) CD8+ T cells, (B) naïve CD4+ T cells, (C) activated memory CD4+ T cells, (D) resting memory CD4+ T cells, (E) Tregs, (F) γδ T cells, (G) T follicular helper cells, (H) naïve B cells, (I) memory B cells, (J) plasma cells. (K-T) Spearman’s correlation of CEACAM5 expression with infiltration of (K) monocytes, (L) M0 macrophages, (M) M2 macrophages, (N) activated myeloid dendritic cells, (O) resting myeloid dendritic cells, (P) activated NK cells, (Q) resting NK cells, (R) eosinophils, (S) activated mast cells, (T) activated mast cells. [file 12885_2022_10397_MOESM3_ESM.pdf]

Additional file 3

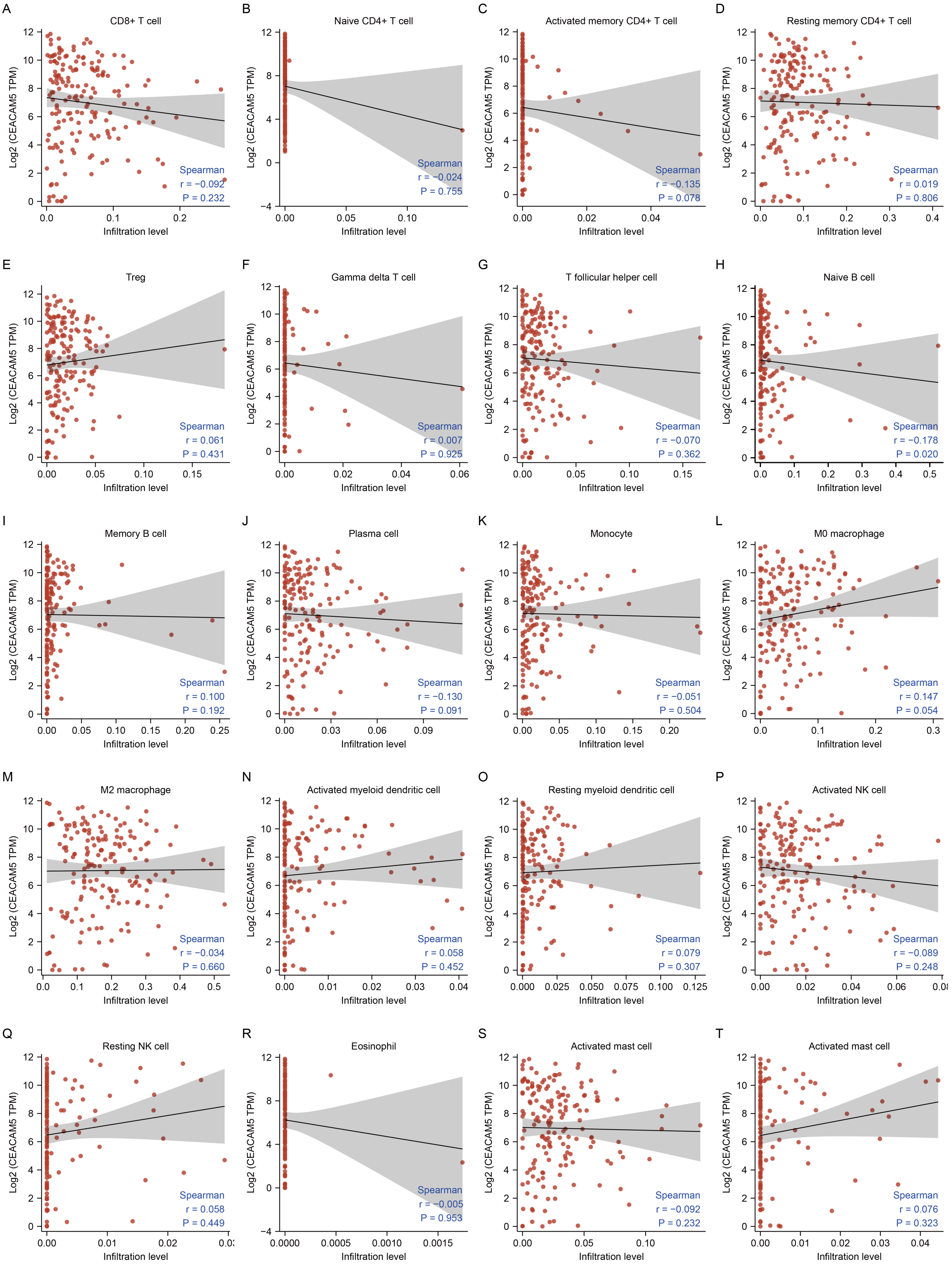

Fig. S3 Association of CEACAM5 expression with immune infiltrates in pancreatic cancer. (**A-J**) Spearman's correlation of CEACAM5 expression with infiltration of (A) CD8^{+} T cells, (B) naïve CD4^{+} T cells, (C) activated memory CD4^{+} T cells, (D) resting memory CD4^{+} T cells, (E) Tregs, (F) γδ T cells, (G) T follicular helper cells, (H) naïve B cells, (I) memory B

cells, (J) plasma cells. (**K-T**) Spearman's correlation of CEACAM5 expression with infiltration of (K) monocytes, (L) M0 macrophages, (M) M2 macrophages, (N) activated myeloid dendritic cells, (O) resting myeloid dendritic cells, (P) activated NK cells, (Q) resting NK cells, (R) eosinophils, (S) activated mast cells, (T) activated mast cells.
