# Supplementary material for: Identification of CEACAM5 as a stemness-related inhibitory immune checkpoint in pancreatic cancer
Source: BMC Cancer. 2022 Dec 9;22:1291. doi: 10.1186/s12885-022-10397-7 (PMC9733357; doi:10.1186/s12885-022-10397-7)
Supplement: Supplementary file 4 — Additional file 4: Fig. S4. Prognostic value of CEACAM expression plus immune infiltration level in patients with pancreatic cancer. (A-J) Kaplan-Meier curves for overall survival split by level of CEACAM5 expression and infiltration level of (A) CD8+ T cells, (B) naïve CD4+ T cells, (C) activated memory CD4+ T cells, (D) resting memory CD4+ T cells, (E) Tregs, (F) γδ T cells, (G) T follicular helper cells, (H) naïve B cells, (I) memory B cells, (J) plasma cells. (K-T) Kaplan-Meier curves for overall survival split by level of CEACAM5 expression and infiltration level of (K) monocytes, (L) M0 macrophages, (M) M2 macrophages, (N) activated myeloid dendritic cells, (O) resting myeloid dendritic cells, (P) activated NK cells, (Q) resting NK cells, (R) eosinophils, (S) activated mast cells, (T) activated mast cells. [file 12885_2022_10397_MOESM4_ESM.pdf]

Additional file 4

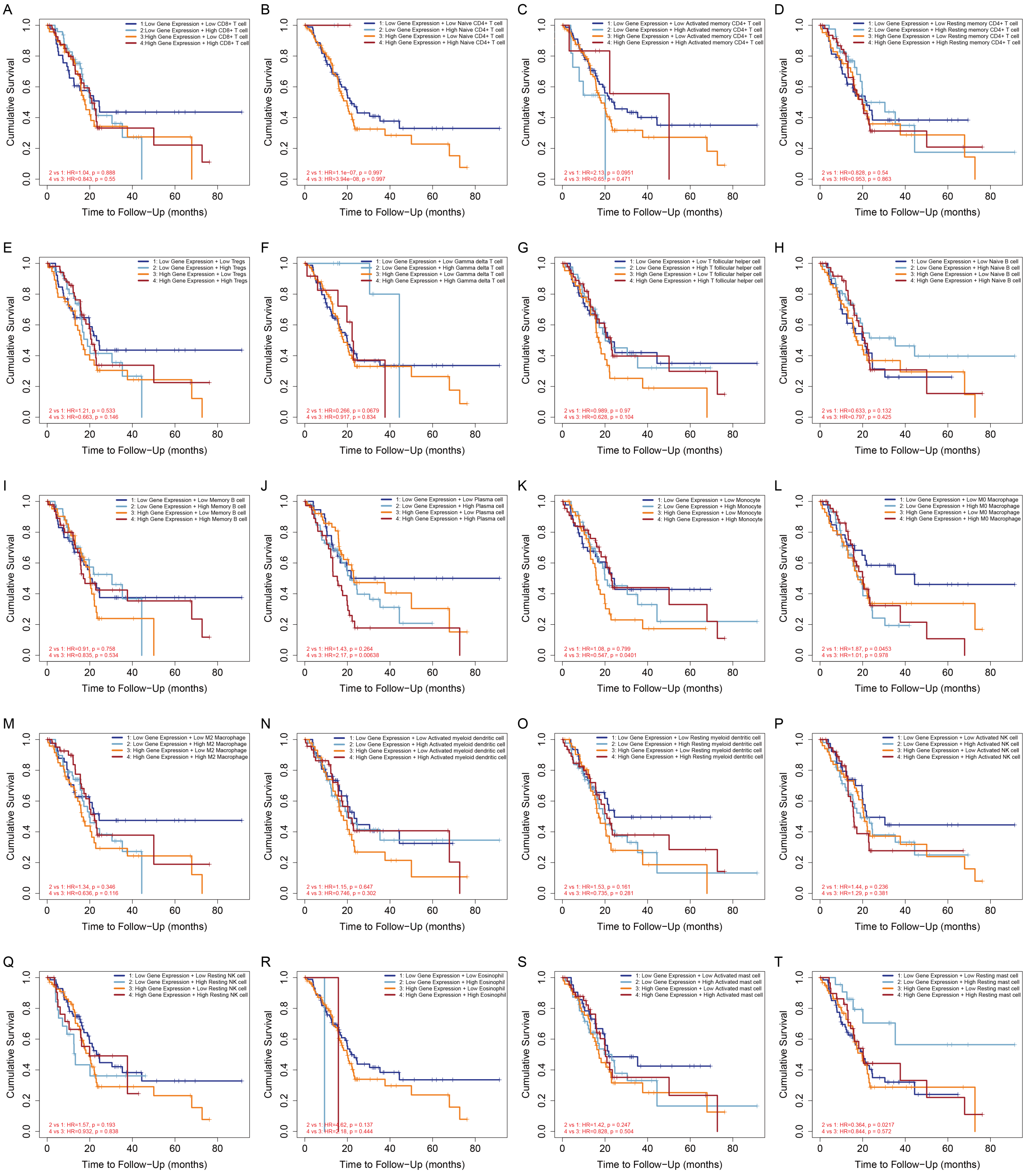

Fig. S4 Prognostic value of CEACAM expression plus immune infiltration level in patients with pancreatic cancer. (**A-J**) Kaplan-Meier curves for overall survival split by level of CEACAM5 expression and infiltration level of (A) $CD8^+$ T cells, (B) naïve $CD4^+$ T cells, (C) activated memory $CD4^+$ T cells, (D) resting memory $CD4^+$ T cells, (E) Tregs, (F) γδ T cells, (G) T follicular helper cells, (H) naïve B cells, (I) memory B cells, (J) plasma cells. (**K-T**) Kaplan-Meier curves for overall survival split by level of CEACAM5 expression and infiltration level of (K) monocytes, (L) M0 macrophages, (M) M2 macrophages, (N) activated myeloid dendritic cells, (O) resting myeloid dendritic cells, (P) activated NK cells, (Q) resting NK cells, (R) eosinophils, (S) activated mast cells, (T) activated mast cells.
