# Supplementary material for: Identification of CEACAM5 as a stemness-related inhibitory immune checkpoint in pancreatic cancer
Source: BMC Cancer. 2022 Dec 9;22:1291. doi: 10.1186/s12885-022-10397-7 (PMC9733357; doi:10.1186/s12885-022-10397-7)
Supplement: Supplementary file 5 — Additional file 5: Fig. S5. Validation using the GSE21501 dataset. (A) Violin plots for CEACAM5 expression in multiple immune subtypes of pancreatic cancer (P=0.0022). C1, wound healing; C2, IFN-γ dominant; C3, inflammatory; C4, lymphocyte depleted; C5, TGF-β dominant. (B) Kaplan-Meier curves for overall survival split by level of CEACAM5 expression and infiltration level of neutrophils in pancreatic cancer. (C) Kaplan-Meier curves for overall survival split by level of CEACAM5 expression and infiltration level of M1 macrophages in pancreatic cancer. [file 12885_2022_10397_MOESM5_ESM.pdf]

Additional file 5

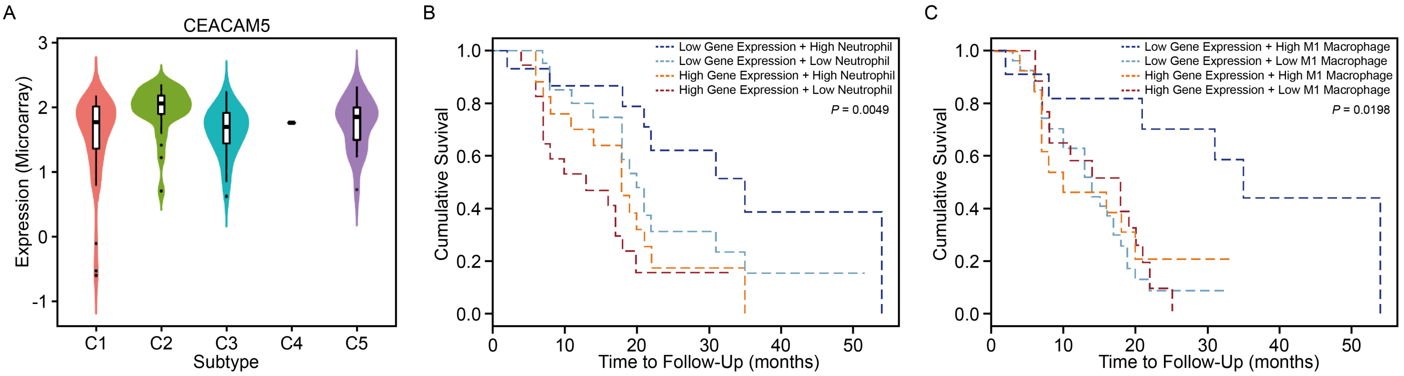

Fig. S5 Validation using the GSE21501 dataset. (**A**) Violin plots for CEACAM5 expression in multiple immune subtypes of pancreatic cancer (*P*=0.0022). C1, wound healing; C2, IFN-γ dominant; C3, inflammatory; C4, lymphocyte depleted; C5, TGF-β dominant. (**B**) Kaplan-Meier curves for overall survival split by level of CEACAM5 expression and infiltration level of neutrophils in pancreatic cancer. (**C**) Kaplan-Meier curves for overall survival split by level of CEACAM5 expression and infiltration level of M1 macrophages in pancreatic cancer.
